# Postoperative complications after cataract surgery with and without concurrent minimally invasive glaucoma surgery in patients with primary open angle glaucoma: a comparative risk analysis

**DOI:** 10.3389/fopht.2026.1830822

**Published:** 2026-06-26

**Authors:** Sinan Ersan, Abdullah Virk, Daniel Zhu, Charles Zhang, Rebecca Zheng Li, Karen M. Allison

**Affiliations:** 1 Jacobs School of Medicine and Biomedical Sciences, University of Buffalo, Buffalo, NY, United States; 2 College of Medicine - Phoenix, University of Arizona, Phoenix, AZ, United States; 3 Department of Ophthalmology, Northwell Health Eye Institute, Great Neck, NY, United States; 4 Department of Ophthalmology, Manhattan Eye, Ear, and Throat Hospital, New York, NY, United States; 5 Bascom Palmer Eye Institute, University of Miami Miller School of Medicine, Miami, FL, United States; 6 Department of Ophthalmology, Ross Eye Institute, SUNY Buffalo, Buffalo, NY, United States; 7 School of Medicine and Dentistry, University of Rochester, Rochester, NY, United States; 8 Department of Ophthalmology, Flaum Eye Institute, University of Rochester Medical Center, Rochester, NY, United States

**Keywords:** cataract surgery, complications, glaucoma surgery, MIGS, primary open-angle glaucoma

## Abstract

**Background:**

Primary open-angle glaucoma (POAG) is the most common form of glaucoma and a leading cause of irreversible blindness worldwide. Treatment focuses on lowering intraocular pressure (IOP), often through cataract extraction with intraocular lens implantation (CE/IOL) alone or combined with minimally invasive glaucoma surgery (MIGS). However, comparative postoperative complication risks between these approaches remain unclear.

**Methods:**

This retrospective cohort study utilized the TriNetX US Collaborative Network to identify all adults (ages ≥18 years) with a diagnosis of POAG who underwent CE/IOL with or without concurrent MIGS between 2006 and 2026. The cumulative postoperative incidence of hyphema, cystoid macular edema (CME), retinal detachment (RD), and endophthalmitis were evaluated at four time intervals up to 90 days postoperatively. Propensity score matching was used to balance baseline characteristics and reduce confounding. Outcomes were compared using relative risks with 95% confidence intervals, and *P*-values were calculated using chi-square tests.

**Results:**

After propensity score matching, each routine cataract surgery cohort (with and without MIGS) comprised 7, 998 patients. Patients undergoing CE/IOL with MIGS had a significantly higher rate of hyphema compared with patients undergoing CE/IOL alone at all reported postoperative time points, with a cumulative incidence of 1.19% versus 0.15% at 1–90 days after surgery, respectively (P<0.0001). Conversely, the cumulative incidence of CME (2.585% vs 2.376%, *P* = 0.3993), RD (0.215% vs 0.139%, *P* = 0.2566) and endophthalmitis (0.276% vs 0.15%, *P* = 0.0862) at 1–90 days postoperatively at 1-90 days postoperatively were not statistically different between groups. Similar associations were observed among patients undergoing routine or complex CE/IOL combined with MIGS compared with those undergoing routine or complex CE/IOL alone with respect to hyphema, CME, and RD.

**Conclusions:**

In this large retrospective cohort study, combined CE/IOL with MIGS was associated with a significantly increased risk of postoperative hyphema, while rates of CME, RD, and endophthalmitis remained comparable to CE/IOL alone.

## Introduction

Glaucoma, the leading cause of irreversible blindness worldwide, is a group of optic neuropathies characterized by progressive damage to the optic nerve and gradual loss of visual fields (1, 2). In 2020, an estimated 76 million people worldwide were diagnosed with glaucoma, and this number is projected to rise to 112 million by 2040 as populations age (2, 3). Of these 76 million people, 3.61 million were blind and 4.14 million were visually impaired, underscoring the significant global impact of glaucoma (4).

Management of glaucoma includes both medical and surgical approaches aimed at reducing intraocular pressure (IOP), the only known modifiable risk factor for the disease (2). Cataract extraction with intraocular lens implantation (CE/IOL) is among the most frequently performed medical procedures and has become an important component of glaucoma management. Phacoemulsification with intraocular lens implantation results in modest but sustained IOP reduction, with studies demonstrating decreases ranging from 4.1 to 8.5 mmHg in patients with open-angle glaucoma (5–7). The IOP-lowering effect is greater in eyes with higher preoperative IOP levels and can persist for several years (5, 8). The IOP-lowering effect of CE/IOL is thought to result from structural changes in the anterior segment, including increased anterior chamber depth and angle opening distance, along with potential improvements in aqueous humor dynamics involving the trabecular meshwork and ciliary body (9, 10).

Minimally invasive glaucoma surgery (MIGS) represents a newer surgical approach for IOP reduction in patients with glaucoma (11). MIGS procedures enhance aqueous drainage through various mechanisms, including, but not limited to, trabecular meshwork bypass with stents (iStent, Hydrus, OMNI), trabecular meshwork excision or ablation (Trabectome, goniotomy), Schlemm’s canal dilation (canaloplasty), or reduction of aqueous production (endocyclophotocoagulation) (1, 2, 8, 11). These procedures offer a favorable safety profile compared to traditional glaucoma surgery such as trabeculectomy, though with more modest IOP reduction, typically achieving postoperative pressures in the middle to upper teens (8, 11).

The combination of CE/IOL with MIGS has become increasingly common, as many MIGS devices are FDA-approved for concurrent use with phacoemulsification (8). Randomized controlled trials have shown that adding trabecular MIGS procedures to routine CE/IOL provides an additional 1.6 to 2.3 mmHg IOP reduction over CE/IOL alone at 2 years, representing an additional 3.8% to 8.9% IOP reduction (12). Approximately two-thirds of the reduction in IOP and medication burden observed in combined procedures is attributable to cataract extraction, with one-third related to the MIGS device (11). Furthermore, combined procedures significantly increase the likelihood of patients achieving medication-free status, with sustained drop-free status at 24 months with addition of Hydrus (RR, 1.6; 95% CI, 1.4 to 1.9) (13).

Despite the effectiveness of combined CE/IOL and MIGS in addressing medication burden and IOP control, there remains a paucity of research comparing the adverse events profiles of CE/IOL alone and in combination with MIGS. The current study aims to address this gap by utilizing a large database to retrospectively compare postoperative complication profiles between these treatment approaches in patients with primary open angle glaucoma.

## Methods

This retrospective cohort study utilized the TriNetX US Collaborative Network, a federated database containing de-identified electronic health record data from a wide range of healthcare organizations around the United States (14). This study was deemed exempt from institutional board review due to the exclusive usage of de-identified, HIPAA-compliant data. The study was performed in compliance with the ethical standards outlined in the Declaration of Helsinki and the World Medical Association’s Declaration of Taipei for the responsible use of health databases (15). Furthermore, it was conducted in accordance with the Strengthening the Reporting of Observational Studies in Epidemiology (STROBE) reporting guidelines for cohort studies (16, 17).

### Cohort definition

The study period encompassed all events between May 14, 2006 to May 14, 2026. A total of 47, 025 patients with primary open-angle glaucoma (POAG; H40.11) who underwent cataract surgery were identified. Patients with a history of injury to the eye or orbit (International Classification of Diseases, 10th Revision [ICD-10] S05), another subtype of glaucoma (ICD-10 H40.13, H40.14, H40.2, H40.3, H40.4, H40.5, H40.6, H40.8, H42), and those with prior glaucoma surgery (Current Procedural Terminology (CPT) codes 0191T, 0253T, 0449T, 0450T, 0474T, 65850, 66170, 66172, 66179, 66180, 66183, 66184, 66185, 66710, 66711, and 67225) were excluded from each cohort. **Supplementary Table 1** lists all codes utilized in cohort selection. A total of six cohorts were established in the present study, and three comparative analyses were conducted based on the specific type of cataract surgery performed.

1. Routine Cataract Surgery: Adult patients (ages ≥18 years) with a diagnosis of primary open-angle glaucoma who underwent routine cataract surgery with or without concurrent minimally invasive glaucoma surgery (MIGS) were identified and stratified into two cohorts:
  1. Routine CE/IOL (CPT 66984) without concurrent MIGS (CPT codes 0191T, 0376T, 0671T, 65820, 66174, and 66175; or combination CE/IOL + MIGS code 66991), and
  2. Routine CE/IOL performed concurrently with MIGS.
2. Complex Cataract Surgery: Adult patients (ages ≥18 years) with a diagnosis of primary open-angle glaucoma who underwent complex cataract surgery with or without concurrent minimally invasive glaucoma surgery were identified and stratified into two cohorts:
  1. Complex CE/IOL (CPT 66982) without concurrent MIGS (CPT codes 0191T, 0376T, 0671T, 65820, 66174, and 66175; or combination CE/IOL + MIGS code 66989), and
  2. Complex CE/IOL performed concurrently with MIGS.
3. Combined Routine or Complex Cataract Surgery: Adult patients (ages ≥18 years) with a diagnosis of primary open-angle glaucoma who underwent routine or complex cataract surgery with or without concurrent minimally invasive glaucoma surgery were identified and stratified into two cohorts:
  1. Routine or complex CE/IOL (CPT 66984 or 66982) without concurrent MIGS (CPT codes 0191T, 0376T, 0671T, 65820, 66174, and 66175; or combination CE/IOL + MIGS code 66991 or 66989), and
  2. Routine or complex CE/IOL performed concurrently with MIGS.

### Primary outcome measures

Four postoperative outcomes were evaluated in the current study: (1) hyphema (ICD-10 H21.0), (2) cystoid macular edema (CME; ICD-10 H35.35, H59.03 or H35.81), (3) retinal detachment (CPT 67108, 67107, 67110, 67113 or ICD-10 H33.0), and (4) endophthalmitis (ICD-10 H44.1, H44.0 or CPT 67015). **Supplementary Table 2** contains all codes utilized in the selection of outcomes. The cumulative incidence of these outcomes were recorded and analyzed at four separate timepoints including 1–14 days postoperatively, 1–30 days postoperatively, 1–60 days postoperatively, and 1–90 days postoperatively. These outcomes were analyzed for routine cataract surgery cohorts, complex cataract surgery cohorts, and combination cohorts. However, analyses of the complex cataract surgery cohorts yielded insufficient data within the TriNetX platform to support meaningful interpretation and were therefore excluded from the present study.

Secondary analyses evaluated individual MIGS procedures performed with routine cataract surgery to further assess outcomes that differed significantly between cohorts in the primary analysis. However, subgroup analyses were limited by inherent constraints of the TriNetX platform. In accordance with patient privacy protections, outcomes involving 10 or fewer patients in either cohort were omitted and therefore excluded from analysis. As such, secondary analyses were similarly not reported in the current study.

### Statistical analysis

Statistical analyses were performed using TriNetX platform’s built in analytics on May 14th, 2026. Propensity score matching was conducted in a 1:1 ratio to balance demographic characteristics and outcome-associated covariates, including variables known or suspected to influence the outcomes of interest, thereby minimizing confounding between cohorts (5, 18–26). Codes utilized for propensity score matching are provided in **Supplementary Table 3**. Matching employed a greedy nearest-neighbor algorithm with a caliper width of 0.1 pooled standard deviations of the logit of the propensity score (27). After matching, the balancing of cohorts was examined using standardized mean differences for each covariate, with <0.1 considered well-balanced. For matched cohorts, relative risks (RRs) with 95% confidence intervals (CIs) were calculated for each outcome. Patients with a history of the outcome of interest prior to surgery were excluded. Comparisons between groups were performed using chi-square tests for proportions. All statistical tests were two-tailed, and a p-value < 0.05 was considered statistically significant.

## Results

1. Routine Cataract Surgery: Prior to propensity score matching, 8, 031 patients in the CE/IOL + MIGS cohort and 12, 958 patients in the CE/IOL alone cohort met inclusion criteria. Following propensity score matching, each cohort comprised 7, 998 patients. Baseline demographic and clinical characteristics before and after matching are presented in **Table 1**.

**Table 1:** Baseline characteristics of patients with undergoing routine CE/IOL and minimally invasive glaucoma surgery (MIGS) vs routine CE/IOL alone before and after matching.

	Before propensity score matching				After propensity score matching			
Characteristics	Cataract surgery + MIGS (n=8031)	Cataract surgery (n=12958)	*P*-value	SD	Cataract surgery + MIGS (n=7998)	Cataract Surgery (n=7998)	*P*-value	SD
Age at index, years, mean (SD)	72.1 (7.83)	70.9 (8.23)	<0.0001	0.1438	72.1 (7.81)	72.1 (7.66)	0.9927	0.0001
Sex, N (%)								
Male	3626 (45.15%)	5496 (42.82%)	0.0010	0.0469	3595 (44.949%)	3604 (45.061%)	0.8863	0.0023
Female	4405 (54.85%)	7339 (57.18%)	0.0010	0.0469	4403 (55.051%)	4394 (54.939%)	0.8863	0.0023
Race, N (%)								
White	4712 (58.673%)	7355 (57.304%)	0.0515	0.0277	4707 (58.852%)	4739 (59.252%)	0.6069	0.0081
Black or African American	2186 (27.22%)	3604 (28.079%)	0.1771	0.0192	2180 (27.257%)	2158 (26.982%)	0.6956	0.0062
Asian	281 (3.499%)	602 (4.69%)	<0.0001	0.0601	281 (3.513%)	302 (3.776%)	0.3756	0.0140
Native Hawaiian or Other Pacific Islander	12 (0.149%)	35 (0.273%)	0.0676	0.0269	12 (0.15%)	10 (0.125%)	0.6696	0.0067
American Indian or Alaskan Native	36 (0.448%)	51 (0.397%)	0.5787	0.0078	34 (0.425%)	33 (0.413%)	0.9026	0.0019
Other	305 (3.798%)	486 (3.787%)	0.9669	0.0006	304 (3.801%)	274 (3.426%)	0.2037	0.0201
Comorbidities (ICD-10-CM), N (%)								
Type 2 Diabetes mellitus (E11)	2286 (28.465%)	4659 (36.299%)	<0.0001	0.1680	2286 (28.582%)	2335 (29.195%)	0.3927	0.0135
Myopia (H52.1)	1173 (14.606%)	2069 (16.12%)	0.0033	0.0420	1172 (14.654%)	1081 (13.516%)	0.0386	0.0327
Nicotine Dependence (F17)	741 (9.227%)	1357 (10.573%)	0.0017	0.0451	741 (9.265%)	740 (9.252%)	0.9782	0.0004
Type 1 Diabetes Mellitus (E10)	203 (2.528%)	804 (6.264%)	<0.0001	0.1830	203 (2.538%)	213 (2.663%)	0.6193	0.0079
Retinal Vascular Occlusions (H34)	215 (2.677%)	486 (3.787%)	<0.0001	0.0628	215 (2.538%)	192 (2.401%)	0.2482	0.0183
Puckering of Macula (H35.37)	578 (7.197%)	1051 (8.189%)	0.0094	0.0372	578 (7.227%)	519 (6.489%)	0.0649	0.0292
Retinal Edema (H35.81)	125 (1.556%)	357 (2.781%)	<0.0001	0.0842	125 (1.563%)	111 (1.388%)	0.3586	0.0145
Lattice Degeneration of the Retina (H35.41)	95 (1.183%)	204 (1.589%)	0.0162	0.0348	95 (1.188%)	65 (0.813%)	0.171	0.0377
Degenerative Myopia (H44.2)	89 (1.108%)	216 (1.683%)	0.0008	0.0490	89 (1.113%)	61 (0.763%)	0.0216	0.0363
Cystoid Macular Degeneration (H35.35)	81 (1.009%)	290 (2.259%)	<0.0001	0.0988	81 (1.013%)	78 (0.975%)	0.8110	0.0038
Retinal Detachment with Retinal Break (H33.0)	62 (0.772%)	261 (2.034%)	<0.0001	0.1074	60 (0.75%)	59 (0.738%)	0.9267	0.0015
Human Immunodeficiency Virus (B20)	29 (0.361%)	88 (0.686%)	0.0023	0.0450	29 (0.363%)	34 (0.425%)	0.5279	0.0100
Acute and Subacute Iridocyclitis (H20.0)	28 (0.349%)	84 (0.654%)	0.0033	0.0433	28 (0.35%)	26 (0.325%)	0.7851	0.0043
Chronic Iridocyclitis (H20.1)	18 (0.224%)	56 (0.436%)	0.0121	0.0370	18 (0.225%)	14 (0.175%)	0.4791	0.0112
Chorioretinal Inflammation (H30)	23 (0.286%)	51 (0.397%)	0.1895	0.0190	23 (0.288%)	18 (0.225%)	0.4343	0.0124
Procedures (CPT), N (%)								
Intravitreal Injection of a Pharmacological Agent (67028)	184 (2.291%)	517 (4.028%)	<0.0001	0.0994	184 (2.301%)	158 (1.975%)	0.1553	0.0225
Vitrectomy, Mechanical, Pars Plana Approach (67042, 67036, 67041, 67040, 67039, 67043)	70 (0.872%)	225 (1.753%)	<0.0001	0.0775	70 (0.875%)	66 (0.825%)	0.7305	0.0054
Repair of Retinal Detachment (67108, 67110, 67107, 67112)	30 (0.374%)	120 (0.935%)	<0.0001	0.0702	22 (0.275%)	19 (0.238%)	0.6390	0.0074
Repair of Retinal Detachment with Vitrectomy (67108)	23 (0.286%)	103 (0.802%)	<0.0001	0.0702	22 (0.275%)	19 (0.238%)	0.6390	0.0074
Pneumatic Retinopexy (67110)	10 (0.125%)	22 (0.171%)	0.3397	0.0122	10 (0.125%)	10 (0.125%)	1.0000	<0.0001
Repair of Retinal Detachment with Scleral Buckle (67107)	10 (0.125%)	13 (0.101%)	0.6227	0.0069	10 (0.125%)	10 (0.125%)	1.0000	<0.0001
Repair of complex retinal detachment with vitrectomy and membrane peeling (67113)	11 (0.137%)	70 (0.545%)	<0.0001	0.0701	11 (0.138%)	13 (0.163%)	0.6829	0.0065
Medications (Veterans Affairs Medication Code), N (%)								
Anticoagulants (BL110)	2066 (25.725%)	3565 (27.776%)	0.0012	0.0463	2061 (25.769%)	2032 (25.406%)	0.5993	0.0083

Baseline characteristics of patients who underwent routine cataract surgery with MIGS (Combined) vs routine cataract surgery alone before and after propensity score matching are shown. Pre-matching baseline characteristics are shown for transparency about baseline differences in groups before matching. Patient demographics and comorbidities are presented for both groups. Standardized differences (SD) and *P* values are shown for balance before and after matching. Propensity score matching was performed using a greedy nearest-neighbor approach. All p-values < 0.05 were considered statistically significant.

2. Complex Cataract Surgery: While a complex cataract surgery cohort was successfully generated, downstream comparative analyses were substantially constrained by TriNetX privacy-preservation protocols, which prohibit reporting of outcomes involving 10 or fewer patients within either cohort. This limitation resulted in multiple suppressed outcome measures across postoperative intervals, yielding incomplete and analytically underpowered datasets. Given the inability to perform robust statistical interpretation and the risk of drawing unreliable or biased conclusions from partially suppressed data, these analyses were deemed unsuitable for formal reporting and were therefore excluded from the present study.
3. Routine or Complex Cataract Surgery: Prior to propensity score matching, 9, 092 patients in the CE/IOL + MIGS cohort and 14, 368 patients in the CE/IOL alone cohort met inclusion criteria. Following propensity score matching, each cohort comprised 9, 040 patients. Baseline demographic and clinical characteristics before and after matching are presented in **Table 2**.

**Table 2:** Baseline characteristics of patients with POAG undergoing CE/IOL (routine and complex) with minimally invasive glaucoma surgery (MIGS) vs CE/IOL (routine and complex) alone before and after matching.

	Before propensity score matching				After propensity score matching			
Characteristics	CAT + MIGS (n=9, 092)	Cataract surgery (n=14, 368)	P-value	SD	CAT + MIGS (n=9, 040)	Cataract surgery (n=9, 040)	P-value	SD
Age at index, years, mean (SD)	72.2 (7.89)	70.9 (8.38)	<0.0001	0.1584	72.1 (7.87)	72 (7.87)	0.4629	0.0109
Sex, N (%)								
Male	4, 223 (46.447%)	6, 262 (43.981%)	0.0002	0.0496	4, 177 (46.206%)	4, 243 (46.936%)	0.3251	0.0146
Female	4, 869 (53.553%)	7, 976 (56.019%)	0.0002	0.0496	4, 863 (53.794%)	4, 797 (53.064%)	0.3251	0.0146
Race, N (%)								
White	5275 (58.018%)	8051 (56.546%)	0.0267	0.0298	5269 (58.285%)	5310 (58.739%)	0.536	0.0092
Black or African American	2514 (27.651%)	4150 (29.147%)	0.0136	0.0332	2507 (27.732%)	2526 (27.942%)	0.7526	0.0047
Asian	316 (3.476%)	650 (4.565%)	<0.0001	0.0555	316 (3.496%)	301 (3.33%)	0.5389	0.0091
Native Hawaiian or Other Pacific Islander	14 (0.154%)	39 (0.274%)	0.0606	0.026	14 (0.155%)	18 (0.199%)	0.4791	0.0105
American Indian or Alaskan Native	40 (0.44%)	56 (0.393%)	0.5874	0.0072	39 (0.431%)	37 (0.409%)	0.8182	0.0034
Other	343 (3.773%)	525 (3.687%)	0.7373	0.0045	341 (3.772%)	309 (3.418%)	0.2011	0.019
Comorbidities (ICD-10-CM), N (%)								
Type 2 Diabetes mellitus (E11)	2647 (29.114%)	5265 (36.979%)	<0.0001	0.1678	2647 (29.281%)	2669 (29.524%)	0.7195	0.0053
Myopia (H52.1)	1320 (14.518%)	2260 (15.873%)	0.0051	0.0377	1319 (14.591%)	1206 (13.341%)	0.0153	0.0361
Nicotine Dependence (F17)	884 (9.723%)	1493 (10.486%)	0.0602	0.0253	880 (9.735%)	846 (9.358%)	0.3895	0.0128
Type 1 Diabetes Mellitus (E10)	242 (2.662%)	934 (6.56%)	<0.0001	0.1867	242 (2.677%)	266 (2.942%)	0.2801	0.0161
Retinal Vascular Occlusions (H34)	244 (2.684%)	549 (3.856%)	<0.0001	0.0659	244 (2.699%)	209 (2.312%)	0.0958	0.0248
Puckering of Macula (H35.37)	666 (7.325%)	1144 (8.035%)	0.0481	0.0267	663 (7.334%)	580 (6.416%)	0.0147	0.0363
Retinal Edema (H35.81)	139 (1.529%)	410 (2.88%)	<0.0001	0.0921	139 (1.538%)	120 (1.327%)	0.2344	0.0177
Lattice Degeneration of the Retina (H35.41)	107 (1.177%)	217 (1.524%)	0.0271	0.0301	106 (1.173%)	96 (1.062%)	0.4792	0.0105
Degenerative Myopia (H44.2)	99 (1.089%)	233 (1.636%)	0.0006	0.0472	99 (1.095%)	98 (1.084%)	0.9429	0.0011
Cystoid Macular Degeneration (H35.35)	95 (1.045%)	319 (2.24%)	<0.0001	0.0942	95 (1.051%)	91 (1.007%)	0.7681	0.0044
Retinal Detachment with Retinal Break (H33.0)	71 (0.781%)	300 (2.107%)	<0.0001	0.1113	71 (0.785%)	69 (0.763%)	0.8653	0.0025
Human Immunodeficiency Virus (B20)	31 (0.341%)	105 (0.737%)	0.0001	0.0542	31 (0.343%)	27 (0.299%)	0.5988	0.0078
Acute and Subacute Iridocyclitis (H20.0)	37 (0.407%)	112 (0.787%)	0.0004	0.0493	37 (0.409%)	28 (0.31%)	0.2634	0.0166
Chronic Iridocyclitis (H20.1)	20 (0.22%)	66 (0.464%)	0.0028	0.0417	20 (0.221%)	19 (0.21%)	0.8726	0.0024
Chorioretinal Inflammation (H30)	27 (0.297%)	61 (0.428%)	0.1101	0.0219	27 (0.299%)	27 (0.299%)	1.0000	<0.0001
Procedures (CPT), N (%)								
Intravitreal Injection of a Pharmacological Agent (67028)	214 (2.354%)	605 (4.249%)	<0.0001	0.1062	214 (2.367%)	185 (2.046%)	0.1421	0.0218
Vitrectomy, Mechanical, Pars Plana Approach (67042, 67036, 67041, 67040, 67039, 67043)	77 (0.847%)	249 (1.749%)	<0.0001	0.0798	77 (0.852%)	71 (0.785%)	0.6204	0.0074
Repair of Retinal Detachment (67108, 67110, 67107, 67112)	31 (0.341%)	136 (0.955%)	<0.0001	0.0766	31 (0.343%)	35 (0.387%)	0.6218	0.0073
Repair of Retinal Detachment with Vitrectomy (67108)	23 (0.253%)	116 (0.815%)	<0.0001	0.0771	23 (0.254%)	24 (0.265%)	0.8839	0.0022
Pneumatic Retinopexy (67110)	10 (0.11%)	23 (0.162%)	0.3069	0.014	10 (0.111%)	10 (0.111%)	1.0000	<0.0001
Repair of Retinal Detachment with Scleral Buckle (67107)	10 (0.11%)	16 (0.112%)	0.9575	0.0007	10 (0.111%)	10 (0.111%)	1.0000	<0.0001
Repair of complex retinal detachment with vitrectomy and membrane peeling (67113)	14 (0.154%)	78 (0.548%)	<0.0001	0.0666	14 (0.155%)	15 (0.166%)	0.8526	0.0028
Medications (Veterans Affairs Medication Code), N (%)								
Anticoagulants (BL110)	2350 (25.847%)	4004 (28.122%)	0.0001	0.0513	2342 (25.907%)	2331 (25.785%)	0.8518	0.0028

Baseline characteristics of patients who underwent routine/complex cataract surgery with MIGS (Combined) vs routine/complex cataract surgery alone before and after propensity score matching are shown. Pre-matching baseline characteristics are shown for transparency about baseline differences in groups before matching. Patient demographics and comorbidities are presented for both groups. Standardized differences (SD) and *P* values are shown for balance before and after matching. Propensity score matching was performed using a greedy nearest-neighbor approach. All *P*-values < 0.05 were considered statistically significant.

**Figure 1:**
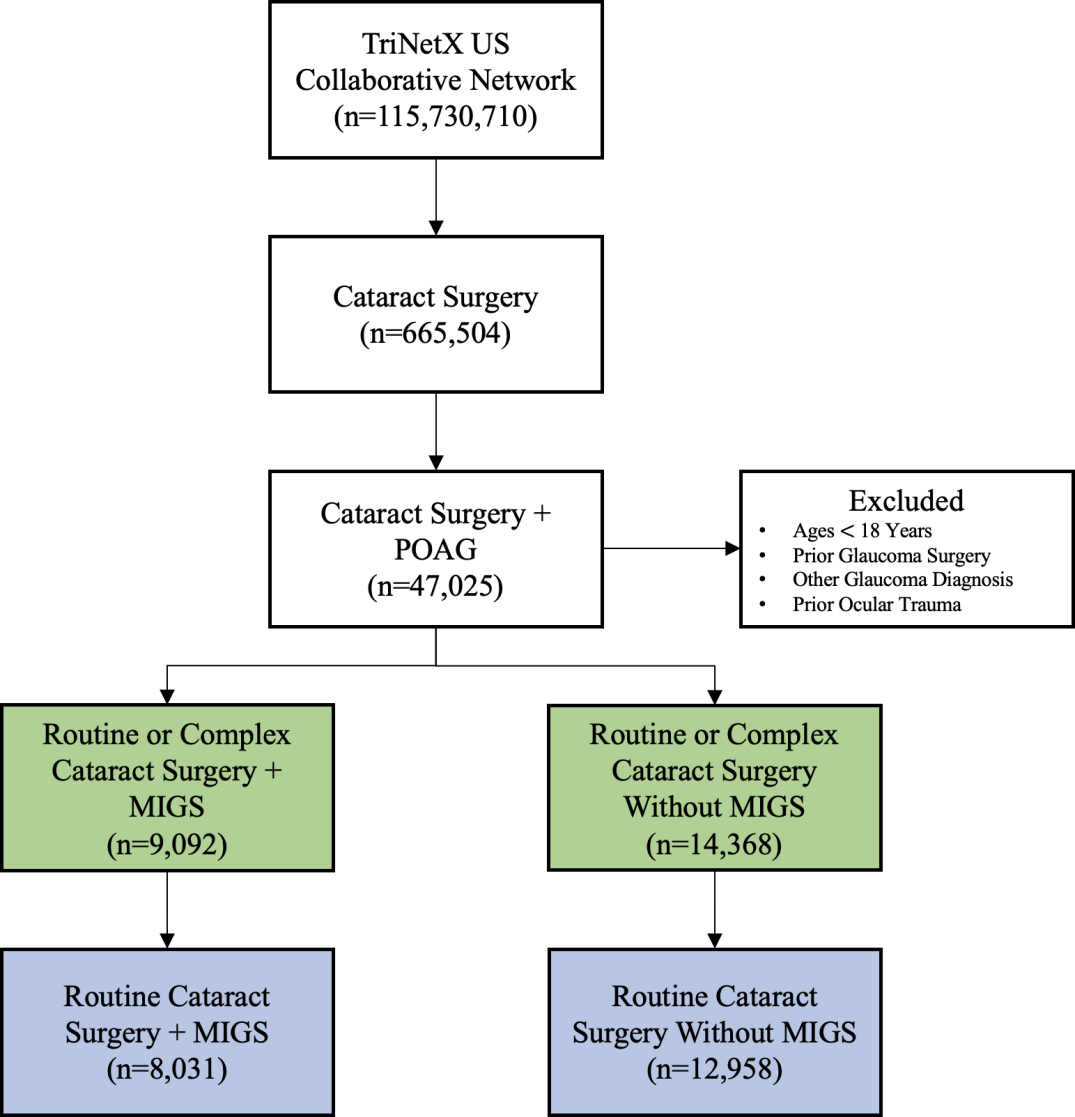
Flowchart illustrating cohort selection process in TriNetX US collaborative network.

**Figure 1** illustrates the cohort selection process in the TriNetX US Collaborative Network.

### Risk of complications

**Figure 2:**
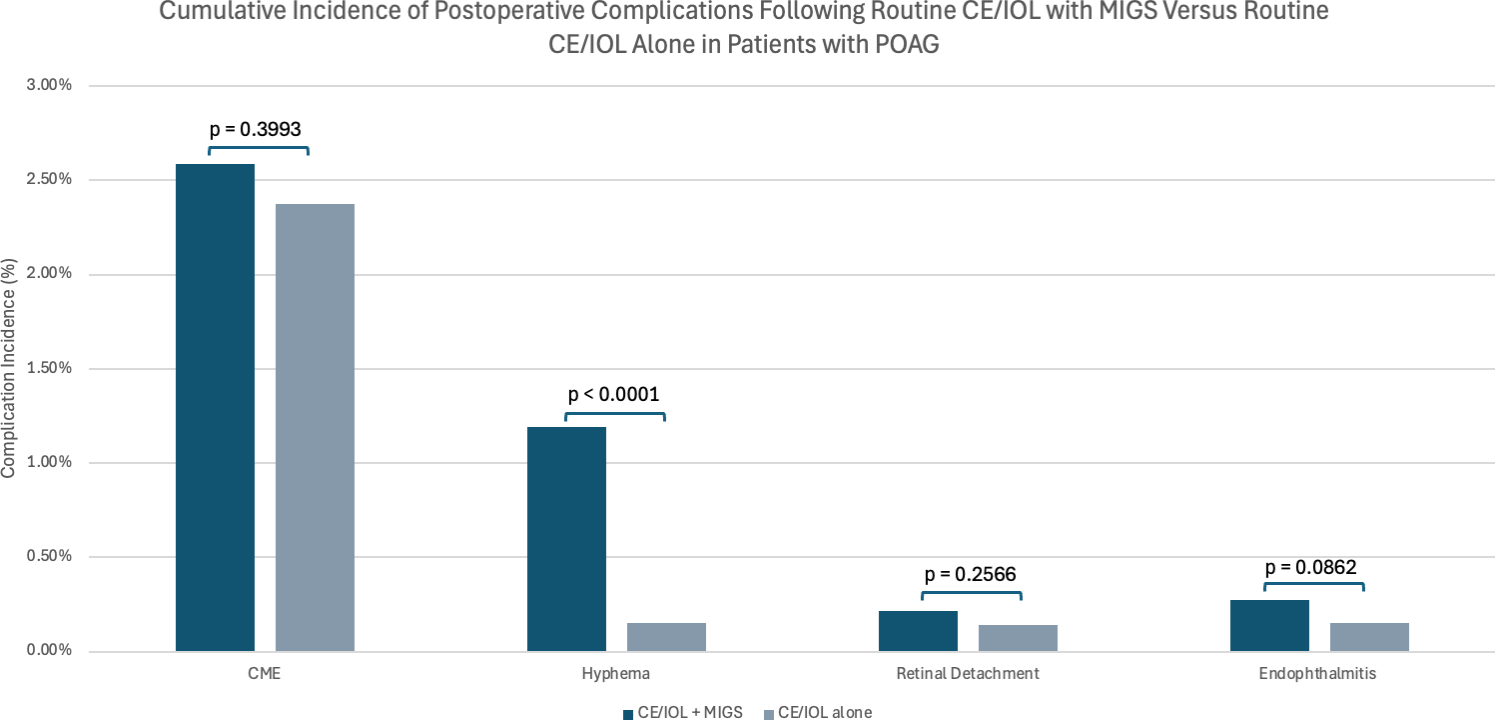
Cumulative incidence of postoperative complications following routine CE/IOL with MIGS versus routine CE/IOL alone in patients with primary open-angle glaucoma (POAG) at postoperative days 1-90.

**Figure 3:**
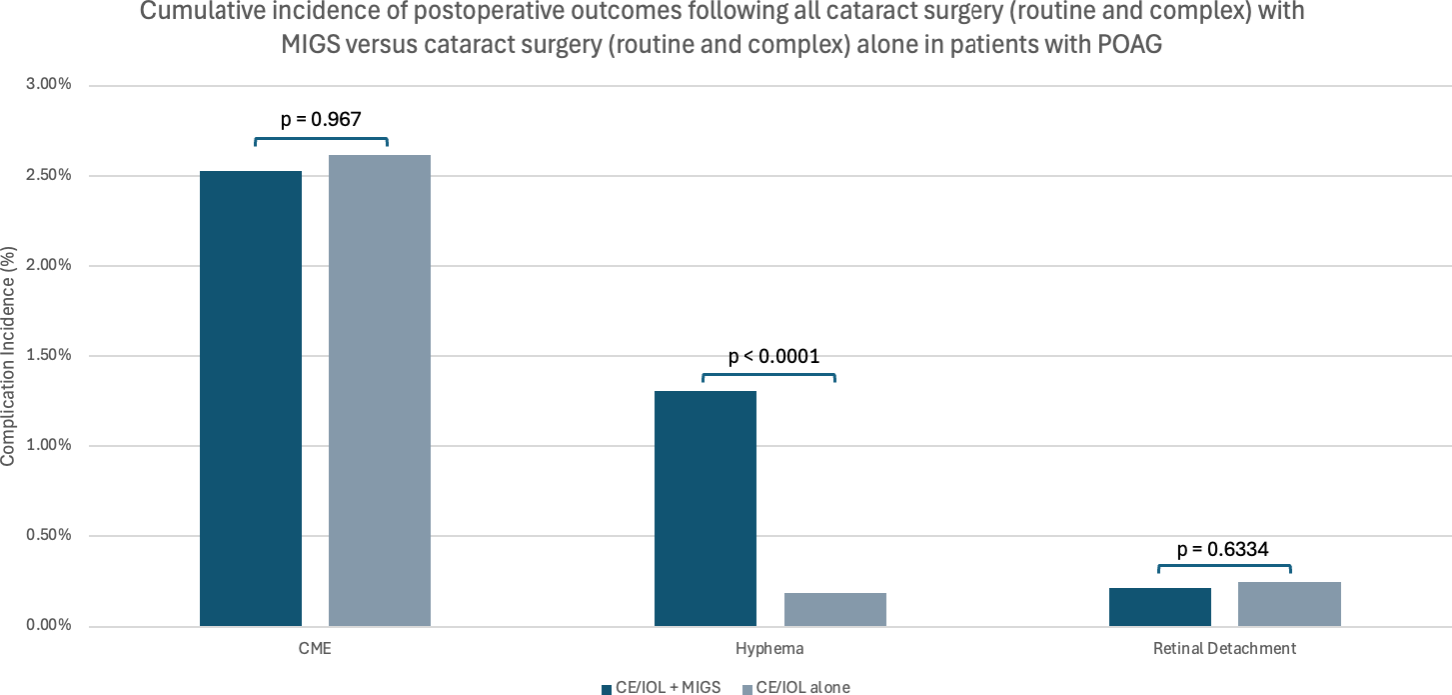
Cumulative incidence of postoperative outcomes following cataract surgery (routine and complex) with MIGS versus cataract surgery (routine and complex) alone in patients with primary open-angle glaucoma (POAG) at postoperative day 1-90.

The cumulative incidence of outcomes at postoperative days 1–14, 1–30, 1–60, and 1–90 was calculated and compared between patients undergoing routine CE/IOL combined with MIGS and those undergoing routine CE/IOL alone (**Table 3**), as well as between patients undergoing CE/IOL (routine or complex) with MIGS and those undergoing CE/IOL (routine or complex) alone (**Table 4**). Overall, patients undergoing combined CE/IOL and MIGS demonstrated significantly higher rates of hyphema across both comparative analyses at all postoperative time intervals for which incidence data were available. Conversely, no significant differences were observed in the cumulative incidence of CME, retinal detachment, or endophthalmitis across either comparative analysis at any postoperative time point for which data were available. **Figures 2**, **3** compare cumulative incidence at 1–90 days between patients who underwent routine CE/IOL with versus without MIGS, and between CE/IOL cases (routine or complex) with versus without MIGS, respectively.

**Table 3:** Cumulative incidence of postoperative complications following routine CE/IOL with MIGS versus routine CE/IOL alone in patients with POAG.

	Time following surgery (days)			
Postoperative Complications	1-14	1-30	1-60	1-90
Hyphema (Combined vs Alone)	NA	NA	1.09% vs 0.138%	1.19% vs 0.15%
RR	NA	NA	7.908	7.916
95% CI	NA	NA	4.227 - 14.796	4.346 - 14.419
*P*-value	NA	NA	<0.0001	<0.0001
Cystoid Macular Edema (Combined vs Alone)	0.218% vs 0.307%	0.755% vs 0.817%	1.984% vs 1.724%	2.585% vs 2.376%
RR	0.71	0.924	1.15	1.088
95% CI	0.382 - 1.32	0.646 - 1.314	0.915 - 1.446	0.894 - 1.325
*P*-value	0.2766	0.6590	0.2290	0.3993
Retinal Detachment (Combined vs Alone)	NA	NA	NA	0.215% vs 0.139%
RR	NA	NA	NA	1.545
95% CI	NA	NA	NA	0.724 - 3.297
*P*-value	NA	NA	NA	0.2566
Endophthalmitis (Combined vs Alone)	NA	NA	NA	0.276% vs 0.15%
RR	NA	NA	NA	1.833
95% CI	NA	NA	NA	0.908 - 3.700
*P*-value	NA	NA	NA	0.0862

Cumulative incidence of postoperative complications following routine cataract surgery with concurrent minimally invasive glaucoma surgery (combined) versus routine cataract surgery alone in patients with primary open-angle glaucoma (POAG). NA values indicate that fewer than 10 patients were diagnosed with the specified condition during the corresponding time interval; therefore, the data were suppressed and unavailable within the TriNetX platform to preserve patient confidentiality.

**Table 4:** Cumulative incidence of postoperative outcomes following all cataract surgery (routine and complex) with MIGS versus cataract surgery (routine and complex) alone in patients with POAG.

	Time following surgery (days)			
Postoperative Complications	1-14	1-30	1-60	1-90
Hyphema (Combined vs Alone)	NA	1.03% vs 0.133%	1.208% vs 0.177%	1.307% vs 0.188%
RR	NA	7.747	6.81	6.939
95% CI	NA	4.250 - 14.124	4.033 - 11.500	4.177 - 11.528
*P*-value	NA	<0.0001	<0.0001	<0.0001
Cystoid Macular Edema (Combined vs Alone)	0.204% vs 0.294%	0.702% vs 0.927%	1.937% vs 1.911%	2.526% vs 2.612%
RR	0.694	0.757	1.014	0.967
95% CI	0.381 - 1.264	0.545 - 1.052	0.821 - 1.251	0.806 - 1.16
*P*-value	0.2295	0.0964	0.8994	0.967
Retinal Detachment (Combined vs Alone)	NA	NA	0.123% vs 0.19%	0.212% vs 0.246%
RR	NA	NA	0.645	0.862
95% CI	NA	NA	0.303 - 1.377	0.467 - 1.591
*P*-value	NA	NA	0.2537	0.6334
Endophthalmitis (Combined vs Alone)	NA	NA	NA	NA
RR	NA	NA	NA	NA
95% CI	NA	NA	NA	NA
*P*-value	NA	NA	NA	NA

Cumulative incidence of postoperative complications following cataract surgery (routine or complex) with concurrent minimally invasive glaucoma surgery (combined) versus routine cataract surgery alone in patients with primary open-angle glaucoma (POAG). NA values indicate that fewer than 10 patients were diagnosed with the specified condition during the corresponding time interval; therefore, the data were suppressed and unavailable within the TriNetX platform to preserve patient confidentiality.

## Discussion

Leveraging real-world data from the TriNetX database, the current study compares the cumulative incidence of postoperative complications between CE/IOL combined with MIGS versus CE/IOL alone. To our knowledge, this is the largest study comparing the cumulative incidence of numerous postoperative complications associated with these surgeries. Our results demonstrated no significant differences in the risk of CME or retinal detachment between groups across both the routine cataract surgery cohort analysis and the combined routine/complex cataract surgery cohort analysis. Furthermore, the cumulative incidence of endophthalmitis was not statistically different between patients undergoing routine cataract surgery. However, both analyses demonstrated that the cumulative incidence of hyphema was significantly higher among patients undergoing combined CE/IOL and MIGS compared with those undergoing CE/IOL alone.

Hyphema, or blood in the anterior chamber, is a possible complication of many intraocular procedures. Granulation tissue formation at the incision site or damage to the uveal vessels are potential mechanisms in which hyphema can form postoperatively (28). Additionally, postoperative hypotony may allow for more blood reflux from any damaged vessels, predisposing to development of hyphema. In CE/IOL, the intraocular lens implant can erode and damage the uveal vessels on the iris, increasing risk of hyphema and Uveitis Glaucoma Hyphema (UGH) syndrome, with one veterans health administration (VHA) study reporting a 0.2% incidence of hyphema within 90 days following cataract surgery (28, 29). This closely resembles the 0.150% - 0.188% cumulative incidence reported in the current study. Studies have also found that hyphema is a common complication of MIGS, with rates varying widely depending on the device and surgical technique. Prior literature highlights that tissue excising procedures, including trabectome, had the highest rates of hyphema, while stenting procedures such as iStent had reported rates between 1.2-1.9% (30–32). MIGS can cause damage to the trabecular meshwork, distal outflow system, or blood vessels adjacent to Schlemm’s canal, resulting in subsequent hyphema which is usually expected to be transient, especially with medical management utilizing topical steroid drops (30, 33, 34). As a result, it is unsurprising that a combination of two surgeries, as seen in the combined CE/IOL and MIGS cohort, resulted in a significantly higher cumulative incidence of hyphema (1.19% - 1.307% at 1–90 days postoperatively, *P* < 0.0001). Unfortunately, the TriNetX database did not have sufficient data for subgroup analysis of specific MIGS devices; therefore, further research is needed to better understand the safety profiles of each individual device and techniques.

Post-operative hyphema can consequently present with increased intraocular pressure (IOP) secondary to obstruction of the trabecular meshwork by platelets, erythrocytes, fibrin, and debris (28). Oftentimes persistent hyphema, if left untreated, can worsen risk of glaucoma and delay visual recovery through mechanisms such as formation of peripheral anterior synechiae (PAS) if hyphema lasts over a week. Although more prevalent in hyphemas of larger volume, PAS are adhesions between the peripheral iris and the corneoscleral angle. The development of PAS can transition POAG to a mixed mechanism glaucoma presentation, which is typically more challenging to manage. Another worrisome complication of hyphema is rebleeding due to the lysis of the original clot, which can further increase the size of the hyphema, raise the IOP, and increase risk for further peripheral anterior synechiae formation (35, 36). Blood reflux can occur with postoperative hypotony, notably when the IOP is lower than the episcleral venous pressure (EVP), which is especially worrisome in MIGS procedures that create a direct communication between the anterior chamber and the episcleral venous circulation. These procedures direct aqueous humor flow into the episcleral venous network based on a pressure gradient of IOP exceeding EVP, but a loss of that gradient in postoperative hypotony will reverse the flow and result in blood spilling into the anterior chamber (37, 38). Nevertheless, some blood reflux is often regarded as a marker of successful creation of a direct connection between the anterior chamber and distal outflow system of aqueous humor (30). Hence, it is important to carefully examine postoperative patients and treat any longstanding hyphema, which could result in further worsening of ocular outcomes.

Endopthalmitis is a feared and visually debilitating complication after any intraocular procedure, increasing risk of permanent vision loss, eye pain, orbital cellulitis, hypotony, swollen lids, and glaucoma amongst a plethora of other symptoms. Although very rare, any procedure that disrupts the integrity of the globe introduces new risk of pathogen invasion, from a variety of species (39). A prior study of Medicare claims data compared the cumulative incidence of postoperative endophthalmitis in patients undergoing combined CE/IOL and MIGS versus CE/IOL alone, with reported rates of 0.176% and 0.13% within 90 following surgery, respectively (40). Although the combined surgery cohort in the Medicare study had a numerically higher incidence, this was not found to be statistically significant on multivariable analysis (40). The cumulative incidence of endophthalmitis in the present study was 0.276% among patients undergoing routine CE/IOL combined with MIGS, compared with 0.15% among patients undergoing CE/IOL alone; however, this difference did not reach statistical significance, consistent with the findings reported by Zafar et al. These results further support the current understanding that MIGS procedures are associated with postoperative infection rates comparable to those observed following cataract surgery alone (41, 42). However, it is likely that the incidence of endophthalmitis reported in the present study may be overestimated, as both diagnostic and procedural codes were utilized for case identification in an effort to mitigate the inherent limitations associated with large retrospective database studies. Still, the discrepancy in magnitude between studies could be due to reporting variability between claims data and EHR based data systems. Mechanistically, combined CE/IOL and MIGS should have similar or only slightly higher rates of endophthalmitis than standalone CE/IOL due to the additional risk of postoperative hypotony, which can allow for clear corneal wound edges to gape open and create a pathway for particle entry (43).

Clinically significant CME occurs in approximately 1%–3% of eyes after routine, uncomplicated phacoemulsification cataract surgery, with large registry data from the AAO IRIS Registry reporting an incidence as low as 0.8% (5, 44). This aligns with the 2.376%-2.612% cumulative incidence of CME observed in the present study at 1–90 days postoperatively. However, this estimate may overstate the true postoperative incidence, as cases of CME identified within the first 30 days following surgery may not be directly attributable to the surgical intervention itself. Nonetheless, the cumulative incidence was calculated at 1–90 days postoperatively, rather than 30–90 days postoperative period, based on reports from prior studies (44). The available evidence suggests that combining MIGS with cataract surgery does not significantly increase the risk of CME compared to cataract surgery alone. In a comparative study of 360 eyes, Schaub et al. found that the incidence of pseudophakic CME was 5.2% after combined trabecular aspiration, 6.7% after ab interno trabeculotomy, and 6.8% after cataract surgery alone, with no statistically significant difference between groups (P=0.676) (45). Similarly, studies of iStent and Hydrus microstent implantation combined with phacoemulsification have reported low rates of CME, comparable to those seen with phacoemulsification alone (46, 47). These findings strongly align with the absence of a statistical difference seen between cohorts in the current study. However, the modality of CME detection used can impact the reported incidence and introduce variability amongst studies. Ophthalmologists who routinely use OCT or fluorescein angiography may detect CME at substantially higher rates than those relying on clinical diagnosis alone. This creates inherent ascertainment bias when comparing CME rates across facilities or providers. However, this bias is likely present among all cohorts.

The risk of retinal detachment following CE/IOL remains low, with one study estimating the risk to be about 0.07% at 3 months postoperatively, increasing to 0.30% within 7 years after surgery (48). The AAO Preferred Practice Pattern cites overall retinal detachment rates of 0.26% - 4% across all time points, with an elevated risk persisting for up to 20 years following surgery (5). The cumulative incidence of 0.139% - 0.246% observed in the current study is higher than the 0.07% reported by Petousis et al., but is lower than the overall lifetime retinal detachment rate reported in preferred practice pattern manuscript. Detection of postoperative retinal detachment in the present study relied on both procedural and diagnostic coding methodologies. While patients with preexisting retinal detachment were excluded and rigorous propensity score matching was performed, limitations inherent to retrospective database analyses raise the possibility that the calculated incidence may overestimate the true occurrence of postoperative retinal detachment. Regarding the addition of MIGS to CE/IOL, we are unaware of a dedicated study that has specifically examined and compared the short-term rates of retinal detachment in these cohorts. The available safety data from clinical trials and reviews of trabecular bypass stents (iStent, Hydrus) and other ab interno MIGS devices report predominantly transient, self-limited complications such as hyphema and IOP spikes, without a signal for increased risk of retinal detachment at any time point (38). These findings align closely with the absence of a statistically significant difference in rate of retinal detachment between the two cohorts in the current study. Nonetheless, it is important to recognize the substantial heterogeneity within the combined CE/IOL and MIGS cohort, as the associated risk profile may differ according to the specific MIGS device or procedure utilized. Consequently, future investigations should evaluate postoperative risk outcomes on a procedure-specific basis to better delineate the safety profile of individual MIGS interventions.

This study has several limitations that should be considered. First, given that TriNetX is an observational database, causal inferences are limited. Due to reliance on medical and procedural codes, certain inconsistencies in coding practices within different healthcare organizations can lead to under or overrepresentation of patients (49, 50). However, in order to minimize inconsistencies in data, the current study utilized propensity score matching and strict criteria in cohort selection. Second, the TriNetX database is unable to provide information regarding the laterality of the eyes analyzed; as a result, this may also influence the overall results, although this would likely affect the outcomes of both cohorts similarly. Third, the TriNetX database does not provide sufficient clinical and postoperative medication data, which creates a core limitation due to complication rates being directly influenced by postoperative treatment regimens. As a result of the lack of data on clinical confounders such as glaucoma severity, baseline intraocular pressure, and treatment regimen, these findings reflect associations in matched observational cohorts rather than conclusive differences. Lastly, specific postoperative timeframes for endophthalmitis and retinal detachment, as well as subgroup analyses by individual MIGS procedure, could not be evaluated due to inherent limitations of the TriNetX network. Specifically, TriNetX does not report patient counts for diagnoses or outcomes when the total number is less than or equal to 10.

## Conclusions

This study demonstrates both overlapping and distinct patterns in cumulative postoperative complication rates between CE/IOL combined with MIGS and CE/IOL alone. Although both procedures have acceptable safety profiles consistent with the current literature, CE/IOL combined with MIGS was associated with a higher incidence of hyphema. Still, the management of patients with ocular hypertension or mild-to-moderate glaucoma with CE/IOL alone or CE/IOL and MIGS can yield favorable results, particularly given the efficacy and safety profiles of MIGS devices. With the growing usage of MIGS in conjunction with CE/IOL, it remains critical to continue analyzing efficacy and complications to better understand its overall safety profile and long-term effectiveness. As surgical techniques continue to evolve, with varying combinations of devices and procedures being utilized, continued evaluation of real-world outcomes and efficacy measures will help optimize surgical decision-making and improve overall patient outcomes. Future studies should further investigate the safety profiles of specific MIGS devices and surgical techniques.

## Data Availability

The data used in this study was analyzed using the TriNetX built-in analytics and are available to reviewers upon request, subject to approval from TriNetX and the corresponding authors institutional policies.
