# Supplementary material for: Postoperative complications after cataract surgery with and without concurrent minimally invasive glaucoma surgery in patients with primary open angle glaucoma: a comparative risk analysis
Source: Front Ophthalmol (Lausanne). 2026 Jun 26;6:1830822. doi: 10.3389/fopht.2026.1830822 (PMC13349877; doi:10.3389/fopht.2026.1830822)
Supplement: Supplementary file 1 [file DataSheet1.docx]

**Supplemental Table 1.** International Classification of Disease, 10^th^ Revision, Clinical Modification (ICD-10-CM) and Current Procedural Terminology (CPT) Codes Used for Cohort Selection

| **ICD-10-CM Code** | **Condition** |
| --- | --- |
| H40.11 | Primary open angle glaucoma |
| S05 | Injury to the eye or orbit |
| H40.13 | Pigmentary glaucoma |
| H40.14 | Capsular glaucoma with pseudoexfoliation of lens |
| H40.2 | Primary angle-closure glaucoma |
| H40.3 | Glaucoma secondary to eye trauma |
| H40.4 | Glaucoma secondary to eye inflammation |
| H40.5 | Glaucoma secondary to other eye disorders |
| H40.6 | Glaucoma secondary to drugs |
| H40.8 | Other specified glaucoma |
| H42 | Glaucoma in diseases classified elsewhere |
| **CPT Code** | **Procedure/Surgery** |
| 66984 | Routine cataract extraction with IOL implantation |
| 66982 | Complex cataract extraction with IOL implantation |
| 66989 | Complex extracapsular cataract removal with IOL insertion + MIGS device (1 or more), internal approach |
| 66991 | Non-complex extracapsular cataract removal with IOL insertion + MIGS device (1 or more), internal approach |
| 0191T | Trabecular micro-bypass stent insertion |
| 0253T | Suprachoroidal insertion of anterior segment aqueous drainage device |
| 0376T | Insertion of anterior segment aqueous drainage device into the suprachoroidal space (internal approach) |
| 0449T | Transscleral cyclophotocoagulation |
| 0450T | Laser or light-based cyclodestruction procedure |
| 0474T | Trabecular micro-bypass stent revision/removal |
| 0671T | Insertion of anterior segment aqueous drainage device (internal approach) |
| 65820 | Goniotomy |
| 65850 | Trabeculectomy |
| 66170 | Implantation of aqueous shunt (without graft) |
| 66172 | Revision or repair of aqueous shunt |
| 66174 | Transluminal dilation of aqueous outflow canal (without retention of device or stent) |
| 66175 | Transluminal dilation of aqueous outflow canal (with retention of device or stent) |
| 66179 | Insertion of anterior segment aqueous drainage device (internal approach) |
| 66180 | Implantation of aqueous shunt (with graft) |
| 66183 | Revision or repositioning of aqueous shunt |
| 66184 | Removal of aqueous shunt |
| 66185 | Removal and replacement of aqueous shunt |
| 66710 | Ciliary body destruction (cyclodiathermy/cryotherapy) |
| 66711 | Cyclophotocoagulation (laser) |
| 67225 | Endoscopic/transscleral cyclophotocoagulation |

**Supplemental Table 2.** International Classification of Disease, 10^th^ Revision, Clinical Modification (ICD-10-CM) and Current Procedural Terminology (CPT Codes Used for Postoperative Outcomes

| **Outcome** | **ICD-10-CM Codes** | **CPT Codes** |
| --- | --- | --- |
| Hyphema | H21.0 |  |
| Cystoid Macular Edema (CME) | H35.35, H59.03, H35.81 |  |
| Retinal Detachment | H33.0 | 67108, 67107, 67110, 67113 |
| Endophthalmitis | H44.1, H44.0 | 67015 |

**Supplemental Table 3.** International Classification of Disease, 10^th^ Revision, Clinical Modification (ICD-10-CM), Current Procedural Terminology (CPT), and Veterans Affairs Medication Codes Used for Propensity Score Matching

| **ICD-10-CM Code** | **Outcome** |  |
| --- | --- | --- |
| E10 | Type 1 diabetes mellitus |  |
| E11 | Type 2 diabetes mellitus |  |
| H52.1 | Myopia |  |
| F17 | Nicotine dependence |  |
| H34 | Retinal vascular occlusions |  |
| H35.37 | Puckering of the macula |  |
| H35.81 | Retinal edema |  |
| H35.41 | Lattice degeneration of the retina |  |
| H44.2 | Degenerative myopia |  |
| H35.35 | Cystoid macular degeneration |  |
| H33.0 | Retinal detachment with retinal break |  |
| B20 | Human Immunodeficiency Virus |  |
| H20.0 | Acute and subacute iridocyclitis |  |
| H20.1 | Chronic iridocyclitis |  |
| H30 | Chorioretinal inflammation |  |
| **CPT Code** | **Procedure/Surgery** |  |
| 67028 | Intravitreal Injection of a Pharmacological Agent |  |
| 67042, 67036, 67041, 67040, 67039, 67043 | Vitrectomy, Mechanical, Pars Plana Approach |  |
| 67108, 67110, 67107, 67112 | Repair of Retinal Detachment |  |
| 67108 | Repair of Retinal Detachment; with Vitrectomy, any method, including, when performed, air or gas tamponade, focal endolaser photocoagulation, cryotherapy, drainage of subretinal fluid, scleral buckling, and/or removal of lens by same technique |  |
| 67110 | Repair of retinal detachment; by injection of air or other gas (eg, pneumatic retinopexy) |  |
| 67107 | Repair of retinal detachment; scleral buckling (such as lamellar scleral dissection, imbrication or encircling procedure), including, when performed, implant, cryotherapy, and drainage of subretinal fluid |  |
| 67113 | Repair of complex retinal detachment (e.g., proliferative vitreoretinopathy, stage C-1 or greater, diabetic traction retinal detachment, retinopathy of prematurity, retinal tear of giant size, choroidal detachment, or detachment due to penetrating injury), with vitrectomy and membrane peeling, including, when performed, air, gas, or silicone oil tamponade, endolaser photocoagulation, cryotherapy, drainage of subretinal fluid, scleral buckling, and/or removal of lens |  |
| **Veterans Affairs Medication Code** | **Medication** |  |
| BL110 | Anticoagulants |  |
